# Cannabis-Based Nanolipid Formulations for Pain Management

**DOI:** 10.3390/pharmaceutics18070844

**Published:** 2026-07-11

**Authors:** Ana Clara Santiago Bastos, Arissa De Oliveira Sato, Luana Carvalho de Oliveira, Fernanda Nervo Raffin, Túlio F. A. L. Moura, Leandro S. Ferreira, Marco V. Navarro, Lígia Nunes de Morais Ribeiro

**Affiliations:** 1 Department of Pharmacy, Faculty of Pharmacy, Federal University of Rio Grande do Norte, Natal 59012-570, RN, Brazil; clara.santiago.101@ufrn.edu.br (A.C.S.B.); luana.oliveira.436@ufrn.edu.br (L.C.d.O.); fernanda.raffin@ufrn.br (F.N.R.); tulio.moura@ufrn.br (T.F.A.L.M.); leandro.ferreira@ufrn.br (L.S.F.); marconavarro@hotmail.com (M.V.N.); 2 Institute of Biotechnology, Federal University of Uberlândia, Uberlândia 38405-319, MG, Brazil; arissa.sato@ufu.br

**Keywords:** *Cannabis sativa* L., herbal drugs, nanoscale, natural products, cannabinoids, pain

## Abstract

Medicinal cannabis has gained increasing attention from both the scientific community and clinical practice, due to the therapeutic potential of its major phytocannabinoids, particularly cannabidiol (CBD) and Δ9-tetrahydrocannabinol (THC), for pain management. This review compiled and analyzed the available evidence regarding the antinociceptive effects of nanoencapsulated cannabinoids compared to free compounds. The published works have explored some pharmaceutical formulations and administration routes on different acute, chronic and neuropathic pain experimental models. The findings indicated that cannabinoids exhibited promising analgesic effects, while nanoencapsulation could enhance its stability and bioavailability. Despite these advances, the number of reports investigating nanostructured cannabinoid-based systems remains limited, with a predominance of preclinical research. A recurrent lack of structural information and quality control data for such works was also noted. Furthermore, there were not identified any research regarding the nanoencapsulation of full-spectrum cannabis oils or whole cannabis extracts, highlighting a significant gap in the current literature. Overall, nanoencapsulation emerges as a versatile strategy to overcome the intrinsic limitations of cannabinoids and expand its clinical applicability for pain treatment. Nevertheless, further efforts are required to determine standardized methodologies, facilitating the translation of preclinical findings into clinical practice, in order to provide stable, safe, effective and more accessible cannabinoid-based therapies.

## 1. Introduction

Pain is a multifaceted and complex neurosensory condition that involves biological, psychological and social factors [1]. The International Association for the Study of Pain (IASP) defines pain as “an unpleasant sensory and emotional experience associated with actual or potential tissue damage” [2,3]. This neurosensory phenomenon impacts the quality of life and behavior negatively [4]. Pain is classified according to its duration and pathophysiology. Regarding duration, pain is divided into acute or chronic [5]. Acute pain is a biological response to short-term tissue injury, gradually decreasing as the body recovers, is treated or heals. Chronic pain is persistent and long-lasting, extending for more than 3 months and potentially lasting for years [6].

In terms of pathophysiology, IASP classifies pain into nociceptive, neuropathic and nociplastic [7]. Nociceptive pain involves the activation of nociceptors that detect actual or potential harmful stimuli and generate a reflex response. Neuropathic pain results from a lesion or disorder of the central nervous system (CNS) or peripheral nervous system (PNS). Nociplastic pain is defined by altered nociceptive processing in the absence of clear evidence of tissue damage or nervous system injury [5]. Due to its complexity, pain management requires a multifactorial approach. Conventional treatments involve combinations of different drugs, such as nonsteroidal anti-inflammatory drugs (NSAIDs), local anesthetics (LAs) and opioids [2,8]. However, such therapies often show limited efficacy in pain management [4].

In addition, the short duration of action of analgesics and LAs requires several doses, which can result in cardiovascular and neurological toxicity [9]. Similarly, NSAIDs exhibit partial efficacy and limited long-term use due to gastrointestinal, renal and cardiovascular toxicity [2]. Opioids induce severe adverse effects that limit its dosage and treatment duration, including cognitive impairment, physical dependence, addiction, tolerance, respiratory depression, constipation and risk of overdose [1,10].

*Cannabis sativa* L., commonly known as marijuana, ganja, or hemp, is an anemophilous species belonging to the *Cannabaceae* family and the *Cannabis* genus, recognized as one of the oldest plants cultivated by humanity [11]. It is an annual plant, mostly dioecious, occurring occasionally in a monoecious form. In this context, it has a straight, hollow stem, with a height ranging from 0.2 to 6.0 m (depending on the genotype, growing and environmental conditions). Female plants are typically more robust and thicker, whereas male plants tend to be more elongated. Notably, female plants are responsible for producing the inflorescences. The leaves are compound and palmate, oppositely or alternately arranged along the stem, ranging from 3 to 13 leaflets at the base and reducing to 1 to 3 in the apical region [12].

**Figure 1:**
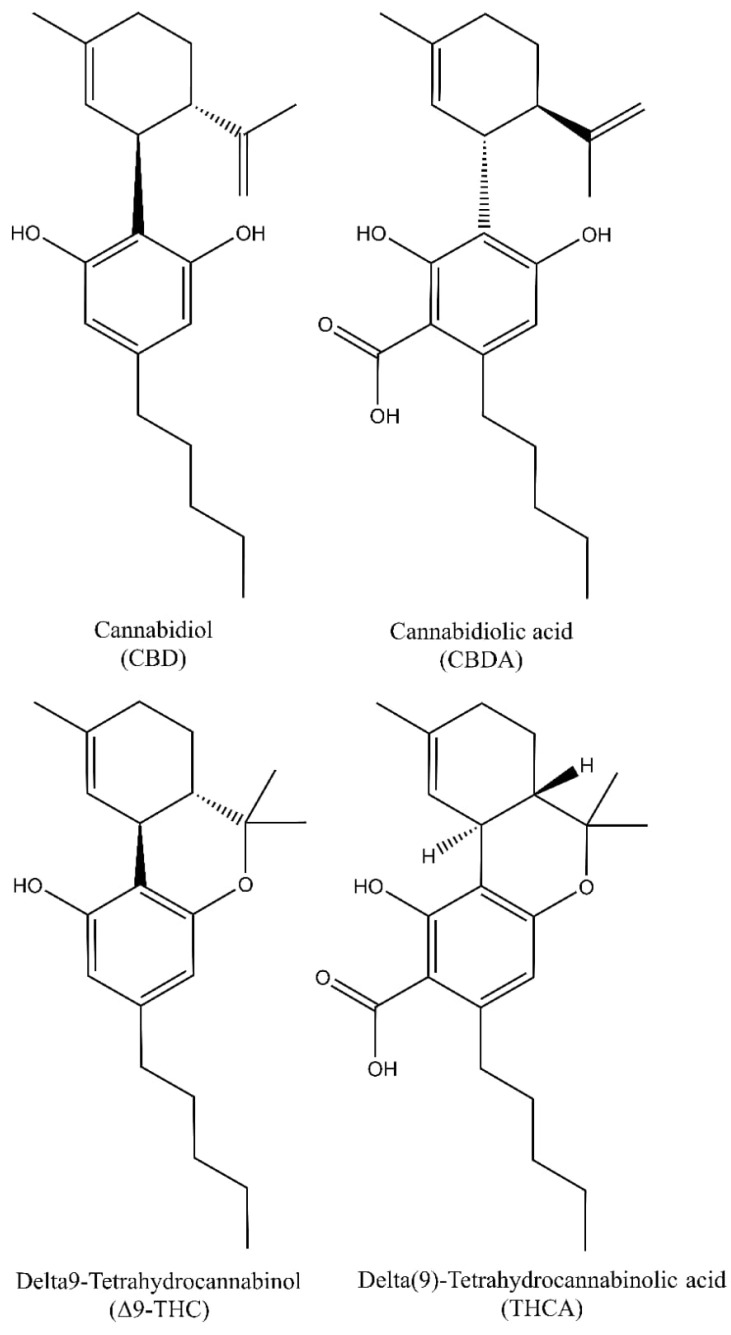
Chemical structures of cannabidiol (CBD), Δ9-tetrahydrocannabinol (Δ9-THC), and its corresponding acids (CBDA and THCA).

*Cannabis* stands out for its high chemical diversity, with over 130 phytocannabinoids already identified, belonging to the class of terpene phenolic compounds [13]. These compounds constitute the secondary metabolites, which are structurally characterized as oxygenated aromatic hydrocarbons, generally consisting of 21 carbon atoms. Among these compounds, Δ9-tetrahydrocannabinol (THC) and cannabidiol (CBD) stand out as the main constituents of plants [14]. Figure 1 displayed the chemical structure of CBD and THC.

THC is recognized as the main psychoactive compound present in *Cannabis*, also exhibiting therapeutic effects, such as analgesic action and appetite stimulation. However, its use may also be associated with adverse effects, including anxiety and episodes of paranoia [15]. This compound is formed through the decarboxylation of tetrahydrocannabinolic acid (THCA). It is a process that occurs during the drying of the plant after harvest. It can be intensified when subjected to heat, as during combustion (smoking), vaporization or cooking preparation [16].

In contrast, CBD has attracted growing interest due to its therapeutic potential in several neurological disorders. In addition, it does not induce psychoactive activity as THC does [17]. This compound is formed through the decarboxylation of cannabidiolic acid (CBDA), a process primarily induced by heating. It belongs to the class of terpenophenols, featuring in its structure a cyclohexene ring, a phenolic ring and a pentyl side chain [18].

**Figure 2:**
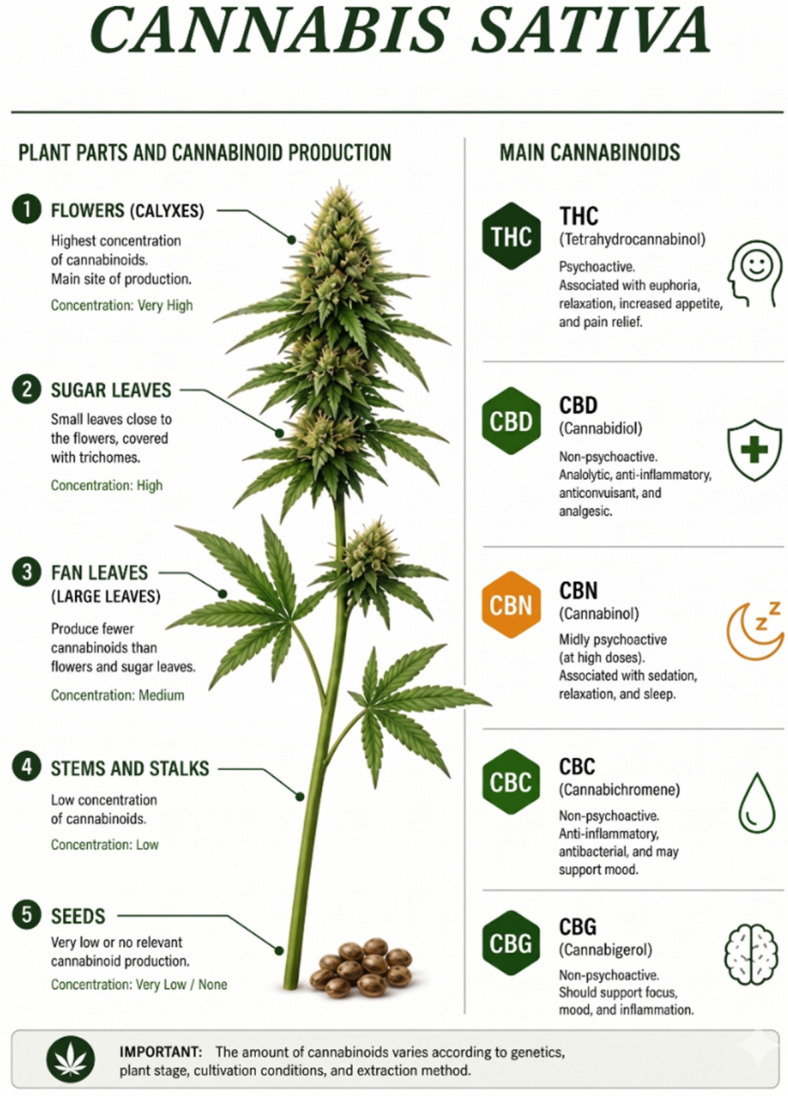
Infographic image of the distribution of cannabinoids from different parts of *Cannabis sativa* and its therapeutic activities.

Other cannabinoids have also been studied given their therapeutic potential. Several novel applications have been investigated. Among them, cannabigerol (CBG) stands out as a non-psychoactive compound with several relevant pharmacological properties, including antibacterial, antifungal and anti-inflammatory activities, increasing its potential for use in multipurpose clinical contexts [19]. Recent investigations have suggested that cannabinol (CBN) can exert antioxidant, anti-inflammatory and neuroprotective effects, reinforcing its pharmacological relevance [20]. Figure 2 illustrated the main cannabinoids found in *Cannabis sativa* and its distribution among different plant structures. The therapeutic effects of cannabinoids in Figure 2 were based on data already reported [21,22].

The biological activity of cannabinoids is associated with its interactions with the endocannabinoid system (ECS), a complex physiological signaling network widely distributed throughout the human body [23]. ECS consists of endogenous mediators, such as anandamide and 2-arachidonoylglycerol (2-AG), as well as its specific receptors (CB1 and CB2) and enzymes involved in biosynthesis and degradation, such as Fatty Acid Amide Hydrolase (FAAH) and Monoacylglycerol lipase (MAGL). The coordinated interaction among these components maintains the endocannabinoid tone, which is essential for the balance of several physiological processes and pathophysiological conditions throughout life [24]. In this communication system, the CB1 and CB2 receptors play essential roles in the transduction of endocannabinoid signals [25].

The CB1 receptor stands out as the most abundant G-protein-coupled receptor in the CNS. It is often described as the primary mediator of the psychoactive properties attributed to *Cannabis* [15]. In addition, CB1 is also present in the peripheral nervous system, particularly in sensory and sympathetic nerve endings, also participating in the regulation of nociception and gastrointestinal motility [26]. Moreover, the CB2 receptor is also a G-protein-coupled receptor. Its expression occurs in immunomodulatory cells, predominantly in glial cells and in the periphery, as well as leukocytes. Furthermore, its expression is dynamically regulated, increasing in response to inflammatory stimuli, particularly in microglia and brain-resident macrophages [27]. CB2 has gained increasing attention due to its role in the modulation of inflammatory, oxidative, immune and apoptotic processes. It is not associated with the adverse psychotropic effects, which generally occur with CB1 activation [28]. 

The nonpolar nature of cannabinoids directs the choice of appropriate methods for its extraction from *Cannabis*. It is generally employed organic solvents such as chloroform and alcohols (methanol and ethanol) or hydrocarbons such as butane and hexane [29]. In addition, decarboxylation process is an important step, once it converts cannabinoid acids into its neutral and bioactive forms, a process that also occurs through heating or during storage [30]. Through these procedures, the *Cannabis sativa* L. extract—the raw material—is obtained, in which the bioactive compounds responsible for the therapeutic effects are concentrated. From this extract, formulations in oil-based vehicles have been developed. However, the term “cannabis oil” lacks scientific precision, as it can refer both to the seed oil, which is free of relevant cannabinoids and to pharmaceutical preparations consisting of extracts diluted in oil [31]. In this context, standardization of extraction, purification and quantification processes become essential to ensure the quality control, stability, safety and efficacy of the cannabis-based processed forms [32].

Cannabis-based extract and oil are rich in cannabinoids that have demonstrated biological activity for conditions such as: chronic pain, CNS disorders, skin diseases, cancer and others [33,34]. Despite its high therapeutic potential, its application is limited by the susceptibility to environmental factors, such as oxygen, light, humidity and temperature, as well as the lipophilic nature of cannabinoids, resulting in its low solubility in physiological media [35].

**Figure 3:**
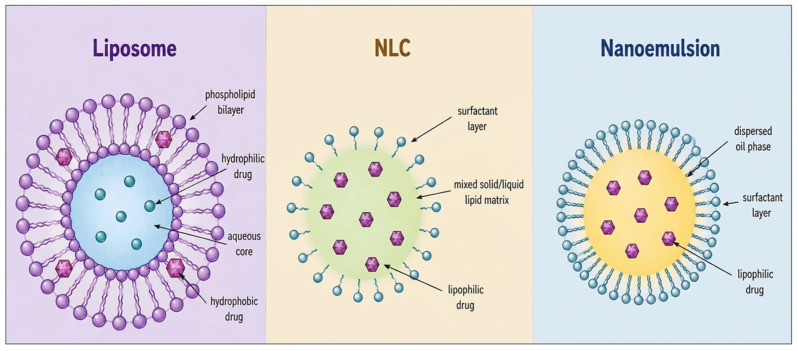
Schematic representation of the main lipid-based nanostructured systems that load cannabinoids: liposomes, nanostructured lipid carriers and nanoemulsions.

The use of nanotechnology processing different nanocolloidal systems is useful to increase the physicochemical stability, solubility and bioavailability of entrapped molecules, resulting in optimized efficacy compared to free forms [36]. In this sense, the lipid nanoparticles can be designed as different nanocarriers with several supramolecular arrangements and modulable biophysical properties, being able to be administered through several routes [37]. All of these carriers have a strong ability to load highly hydrophobic molecules. There are some reports of cannabinoid encapsulation by liposomes (LUV), nanostructured lipid carriers (NLC) and nanoemulsions (NE) for human and veterinary clinical use, as illustrated in Figure 3.

The liposomal system is the pioneering lipid nanocarrier that has already been developed. There are some FDA-approved liposomal drugs, such as Doxil^®^ and Ambisome^®^. LUV are composed of at least one phospholipid bilayer and cholesterol, surrounding an aqueous inner core. It is the most versatile nanocarrier, once it has affinity to encapsulate hydrophilic (aqueous core), hydrophobic (lipid bilayer) and amphiphilic (polar head and acyl chain interface) molecules. There are a lot of works that described LUV encapsulating highly hydrophobic molecules with success [38].

The second generation of lipid nanoparticles, known as NLC, configure the most developed nanocarrier based on natural lipids for multipurpose applications. This success is related to the nature of its structural matrix, which is composed of a blend of solid and liquid lipids, sterically stabilized by surfactants. Thus, natural lipids loaded by NLC exert a dual role in the formulations: structural and bioactive. The natural liquid lipid presence improves the nanoparticle arrangement, given by the formation of structural defections in the solid lipid matrix, which is required for this nanocarrier stability and efficacy [39]. Finally, NE are colloidal systems composed of liquid lipid and stabilized by surfactant and co-surfactant. Such systems have an affinity for loading natural lipids and exhibit long-term stability [40].

In this sense, the development of cannabis-based formulations have to ensure a set of structural parameters to warrant the quality control. A robust analytical method to quantify the cannabinoid upload indexes and the in vitro release profile is paramount. The monitoring of biophysical parameters has to be performed, in order to determine its shelf life. Then, only the systems with the best performance in all the above-mentioned requirements will be submitted to subsequent biological and preclinical assays. The cannabis-based nanodrug development has to include all these steps of analyses to provide a robust, safe and effective nanodrug.

## 2. Application of Cannabis-Based Lipid Nanoparticles for Pain Management

This review presents the main highlights of the potential use of lipid nanostructured systems as a strategy to enhance the antinociceptive effect of cannabinoids. Despite there were a few published works, some reports were detailed regarding the development of LUV, NLC and NE that loaded extract, oil and/or synthetic cannabinoids, aiming at pain management. This is opening new alternatives to overcome the intrinsic limitations of non-encapsulated cannabis-based products for human and other animal uses.

## 3. Application to Human Health

Currently, medicinal cannabis has been extensively investigated, due to the therapeutic potential of THC and CBD. Evidence from literature reviews suggested that these compounds can exert antinociceptive effects in conditions such as chronic pain. However, its intrinsic instability and hydrophobicity limit its efficacy. Therefore, the development of lipid nanoparticles loading cannabinoids is a promisor strategy to enhance its stability, bioavailability and analgesic potential in the treatment of pain in humans. Table 1 summarized the available published works regarding cannabis nanomedicine for human pain management.

A CBD-based NE aimed at intrathecal administration for the treatment of pain in rats. The formulation demonstrated physicochemical stability over 70 days at 4 °C, with particle sizes ranging from 286.8 to 294.1 nm and exhibiting a narrow and homogeneous particle size distribution, as evidenced by polydispersity index (PDI) values close to 0.137, along with zeta potential (ZP) values of −53.5 mV. Regarding safety, the absence of detectable CBD in the liver, spleen, deep cervical lymph nodes, and serum of rats, together with the higher concentration observed in the spinal cord, suggested localized distribution and reduced systemic exposure, confirming the biocompatibility of the formulation. It also showed a pronounced antinociceptive effect in rats [41].

Matarazzo and coworkers [42] developed mucoadhesive (1 mg/mL) NLC/CBD formulations processed as gels for intranasal administration of CBD for neuropathic pain management. The biophysical characterization demonstrated particle size of 177.0 nm and PDI values close to 0.300, indicating a moderate homogeneous particle size distribution. A highly positive ZP value of +41.0 mV was detected, contributing to the mucoadhesive properties, given by the presence of cetylpyridinium chloride excipient in the composition. The formulation also showed encapsulation efficiency (%EE) close to 99.99%, attributed to the high lipophilicity of CBD. The in vivo assays confirmed the improved CBD retention time in the mucosa, with more intense and prolonged antinociceptive effect, observed in mice.

Another recent report developed natural NLC formulations containing 5% (*w*/*v*) CBD with antinociceptive activity [1]. The resultant system exhibited long-term physicochemical stability for 365 days at 25 °C, with size close to 236.3 nm, PDI values of 0.153 and ZP of −32.30 mV even after a year of storage, confirming the shelf-life. Alternative biological models were carried out to elucidate the safety and efficacy of NLC/CBD. The nanotoxicity assays using a chicken embryo model determined a concentration-dependent effect of NLC/CBD, with no nanotoxicity detected in the concentration range from 0.72 to 2.87 mg/mL. Moreover, in the nociceptive activity assessment in the *Drosophila melanogaster* model, NLC/CBD exhibited significant antinociceptive effects from 1.0 mg/mL of CBD.

Another work described a hybrid system composed of LUV containing CBD for transdermal administration [43]. The system composed of deformable LUV contained Tween 80^®^, responsible for increasing the flexibility of the phospholipid bilayer. It showed an %EE of 92.2 ± 3.4% for CBD with a particle size of 81.3 ± 0.2 nm. CBD demonstrated a strong affinity for the lipid bilayer. Such formulation showed the highest skin permeation and dermal retention, promoting a sustained drug release of CBD with a faster onset. In terms of efficacy, the formulation containing 0.2% (*w*/*v*) CBD demonstrated higher therapeutic activity, even at lower concentrations, than those found in commercial formulations (5–10%).

In summary, nanostructured lipid systems significantly modulated the pharmacokinetic and pharmacodynamic profiles of cannabinoids, impacting their antinociceptive efficacy directly. Factors such as particle size, composition and route of administration are critical determinants, making the selection of an appropriate lipid nanocarrier essential for optimizing pain management.

## 4. Application to Veterinary Medicine

Animal welfare is a major concern in veterinary medicine [44]. Different strategies and novel drug therapies have been developed to improve the quality of life of animals [45]. The ECS is widely distributed in most animals, including tissues such as CNS and PNS, gastrointestinal tract, skin and joints [46]. In this context, the therapeutic potential of cannabis-derived compounds has increasingly been investigated in veterinary medicine [44,47].

These phytocannabinoids have been evaluated for different therapeutic purposes in dogs, cats, horses and goats. Among the applications, it has currently found uses for pain control (osteoarticular, oncological and neuropathic pain), epilepsy, oncology and for inflammatory, immune-mediated, cardiovascular, respiratory and dermatologic diseases [48,49]. There are a few works with cannabinoids, mainly CBD, for pain management in animals. In most cases, pure extracts or oil-based cannabinoid are evaluated [50,51,52].

Gamble et al. conducted a randomized, placebo-controlled, double-blinded, cross-over study to evaluate a CBD-in-oil for symptom management of osteoarthritic dogs. The animals received CBD (2 mg/kg) or placebo (olive oil aromatized with anise and peppermint oils) orally, every 12 h for 4 weeks, with a 2-week washout period in between treatments [53]. Each dog was evaluated using the Canine Brief Pain Inventory (CBPI) and the Hudson Activity Scale. Animals treated with CBD showed a significant reduction in pain, as evidenced by lower CBPI scores, along with a significant improvement in physical activity and mobility, reflected by higher scores on the Hudson Activity Scale when compared to the placebo group [53].

Brioschi et al. evaluated a transmucosal administration of CBD in comparison with a multimodal pharmacological treatment for chronic osteoarthritis pain in dogs. The animals were randomly assigned in two groups: treated with CBD oil (2 mg/kg) and not treated with CBD. The measures were performed based on CBPI, pain severity score (PSS), pain interference score (PIS) and quality of life index (QoLI). PSS and PIS were significantly higher in the CBD group than the control group. In addition, QoLI was significantly higher for the CBD group [50].

It is clear that most CBD oil treatments have been administrated orally. In this route, the bioavailability of oily therapies is reduced due to the first-pass effect [47]. Oral CBD administration often requires multiple administrations [54]. To overcome this limitation, strategies to improve bioavailability have been developed, such as lipid-based delivery systems [54,55]. Across a series of works, [54,55,56] evaluated injectable liposomal CBD formulations (InnoCan Pharma, Israel) in dogs with osteoarthritic pain. The liposomal CBD formulation encapsulated synthetic CBD [55]. There are no data regarding PDI or ZP values, nor shelf life.

## 5. Approved Cannabis Products for Biomedical Use

Despite the global dissemination of cannabis-based products, the number of approvals by regulatory agencies remains limited. Such applications are mainly concentrated in rare epilepsies, spasticity associated with multiple sclerosis, management of chemotherapy-related symptoms, and, in some cases, chronic pain and palliative care [57]. Table 2 summarized the cannabis-based products for human and veterinary uses.

India adopted the AYUSH (Ayurveda) system focused on development, education and research to promote holistic well-being. Therefore, a regulated parallel market was created [44]. India follows The Narcotic Drugs and Psychotropic Substances (NDPS; 1984), in which the cannabis flower/resin is forbidden, while leaves and seeds are legalized. Their regulation requires THC < 0.3%, AYUSH licensing and a medical prescription [58]. Standardized pharmaceutical formulations coexist with Ayurvedic cannabis-based products, highlighting a hybrid regulatory model with broad therapeutic diversity [59,60]. This is probably the reason for the dozens of products licensed as Ayurvedic *Cannabis* formulations in the Indian local market, in contrast to other continents where there are a few approved cannabis-containing drug products [61]. It is worth noting that AYUSH products are composed of complex herbal formulations of CBD combined with Ayurvedic herbs and non-standardized extracts, leading to high diversity, but low scientific standardization [62,63] and clinical evidence [61,64,65]. Clasepi was the first CBD-based product approved in India. It is indicated for refractory epilepsy, with THC < 0.1%, equivalent of Epidiolex^®^ [66,67,68]. On the other hand, several cannabis-based products have also been employed for pain management in different countries.

In Brazil, cannabis-based products are regulated under a specific framework (RDC 1012/2026) and are classified as “Cannabis-derived products” rather than fully registered pharmaceuticals. This places Brazil in an intermediate regulatory position among strictly approved pharmaceutical markets (e.g., USA, Europe) and more flexible phytotherapeutic systems (e.g., India). There are a few commercially available drugs containing cannabis in Brazil for the treatment of pain, such as Cannabidiol^®^ (Prati-Donaduzzi), Cannabis Extract^®^ (Promediol) and Cannabis Extract^®^ (Cannabr). Such prices showed significant variability depending on concentration and formulation, ranging from low-cost to high-potency products exceeding $300 per unit. The most of such formulations are composed of combined CBD and THC, being mainly administered as oral oils, indicated for chronic pain and palliative care.

Similarly, Indian products such as Cannavedic^®^ (CannaStrong) and Cannazo^®^ (Vijaya Ambrosia) are also administered as oral oils, prescribed for severe chronic pain, inflammation, edema, insomnia and palliative care. In addition, Europe and Oceania trade Sativex^®^, an oromucosal spray containing CBD and THC that has been approved for spasticity associated with multiple sclerosis. Its off-label use for pain relief in neurological conditions is frequent. These examples demonstrated the growing diversification of cannabinoid-based therapies for pain-related conditions worldwide.

Furthermore, the available data demonstrated that many cannabis-based formulations intended for pain management and other chronic conditions presented high costs, often ranging from hundreds to more than a thousand dollars per month, depending on the formulation and country. These expensive values restrict access for economically vulnerable populations significantly, limiting their broader therapeutic use.

## 6. Perspectives

This review systematized a set of works regarding the antinociceptive potential of free and nanoencapsulated cannabinoids through different experimental pain models. Most works still focus on evaluating these compounds in a free form for oral administration or processed as gels or creams for skin delivery. Cannabis-derived oils and extracts are highly hydrophobic, photosensitive and volatile (terpenes). In addition, the cannabis-based forms, when orally administered, will be submitted to the first-pass effect, which limit its efficacy. Unfortunately, there are a limited number of works of Nanocannabis-based formulations that were molecularly planned to solve such intrinsic limitations. On the other hand, most of the published works have shown a lack of quality control information regarding nanoformulations. The elucidation of its structural organization was also scarce. Still, there was a predominance of preclinical studies, with poor comparisons between free and nanostructured cannabis pharmaceutical forms, which hindered a comprehensive assessment of its therapeutic advantages.

Moreover, the related regulatory issues also present relevant challenges to be overcome. There are different cannabis drug regulation laws among countries, and, to date, there is no specific regulation for the approval of lipid nanodrugs. The lack of well-established regulatory frameworks significantly limits the investigations into the long-term stability, safety, efficacy and the determination of clinical doses of cannabinoid-based therapies in humans and other animals. These conditions delay the progression of these technologies toward clinical use.

The availability of phytocannabinoid products is more restricted in veterinary than in human medicine. In most countries, the regulation to access cannabis for medical purposes is applied specifically to humans, limiting the legal use in other animals. Consequently, most cannabinoid-based products used in veterinary practice are off-label administered, being originally developed for human applications. Despite these regulatory limitations, it was observed a growing number of cannabinoid-based products available in the pet care market. However, many of these products are commonly commercialized as supplements, allowing manufactures to circumvent stricter pharmaceutical regulatory requirements related to quality control, efficacy and safety assessment. In fact, the field of research on veterinary medicinal cannabis remains limited, and many published works have reported conflicted findings with small sample sizes. Notably, to date, only a single commercially available liposomal CBD formulation has been investigated for pain management in animals.

It is clear that the development of nanostructured lipid-based carriers acts as a promising strategy to overcome the intrinsic limitations of cannabinoids. Further works may focus on the design of lipid nanostructured systems, as well as the evaluation of alternative administration routes, such as intranasal and oral transmucosal. Furthermore, strengthening the integration between academic research and pharmaceutical industry is essential to enable to scale up production, ensure regulatory compliance and accelerate clinical translation.

## 7. Conclusions

Medicinal cannabis has attracted increasing scientific and clinical interest, due to the analgesic potential of its major cannabinoids, particularly CBD and THC. Reports suggest that these compounds represent a promising alternative therapy for pain management, especially for chronic and neuropathic conditions.

Although the number of works that investigated nanoencapsulated cannabidiol pain management are scarce, the available evidence demonstrated important physicochemical and pharmacological advantages of these delivery systems, including enhanced stability, sustained release profile, improved bioavailability and optimized efficacy. Notably, no work related to the nanoencapsulation of full-spectrum cannabis oils or cannabis extracts was identified.

Another important observation was the lack of structural and quality control information for the developed formulations. Essential analytical parameters, such as encapsulation efficiency, drug release profile and stability monitoring were most times insufficiently reported or entirely absent, as evidenced in Table 1 and Table 2. These limitations highlight the need for standardization of characterization methods and quality control procedures, in order to ensure the reproducibility and future clinical translation of these systems.

Overall, advances in the nanoencapsulation of cannabinoids and other cannabis-derived products can significantly contribute to the development of stable, safe, effective and cost-effective pain therapies, particularly for chronic and neuropathic pain conditions. The economic viability of these systems represents a key factor for its approval and further potential incorporation into public healthcare systems. Thereby, we are looking to improve access to innovative therapies for acute and chronic pain management.

## Figures and Tables

**Table 1:** Summary of available published works on lipid nanoparticles loading cannabinoids for pain management.

Author/Year	Formulation	Size (nm)	PDI	ZP (mV)	EE (%)	Quality Control, Safety and Efficacy Results
Muresan et al., 2023 [41]	CBD-NE (0.1 g/mL)	286.8–294.1	0.135–0.139	−54.1 to −53.2	NR	Stability for 70 days; local biodistribution in the CNS; absence of peripheral biodistribution (liver, spleen and lymph nodes); potential systemic safety and analgesic action on a rat model.
Matarazzo et al., 2021 [42]	Mucoadhesive NLC/CBD (1 mg/mL)	177.0	0.300	+41.0	99.99	High mucoadhesion, better nasal retention; prolonged release profile; intensified antinociceptive effect on mice model.
de Oliveira Sato et al., 2026 [1]	NLC/CBD (5% *w*/*v*)	236.3	0.153	−32.30	NR	Stability for 1 year; biocompatible on a chicken embryo model; antinociceptive effect on *Drosophila melanogaster*.
Franzè et al., 2022 [43]	Deformable LUV/CBD (0.2% *w*/*v*)	81.3 ± 0.2	NR	NR	92.2	High skin permeation; sustained release profile; reduced dosage; in vivo and ex vivo biocompatibility; improved control on neuropathic pain.

**Table 2:** Cannabis-based products for pain management: prescription status, prices, and applications worldwide.

Continent	Drug	Cannabinoid	Prescription	Price (USD)	Application
North America	Epidiolex	CBD	Epilepsy (Dravet, Lennox–Gastaut, TSC)	U$1050	Human
North America	Marinol	THC (synthetic)	Nausea/emesis (chemotherapy), anorexia (HIV)	U$800–1200/month	Human
North América	Syndros	THC (synthetic)	Nausea/emesis (chemotherapy), anorexia (HIV)	U$1000/month	Human
North America	Cesamet	THC (synthetic)	Refractory nausea and emesis (chemotherapy)	U$500–900/month	Human
Europe	Epidyolex	CBD	Epilepsy (Dravet, Lennox–Gastaut)	U$1000	Human
Europe	Sativex	THC + CBD (extract)	Spasticity in multiple sclerosis	U$600–1200/month	Human
Oceania	Sativex	THC + CBD (extract)	Spaticity (multiple sclerosis)	U$700–1300/month	Human
Oceania	Epidiolex	CBD	Refractory epilepsy	U$1000	Human
Latin America	Epidiolex	CBD	Refractory epilepsy	U$1000	Human
Latin America	Sativex	THC + CBD (extract)	Multiple Sclerosis	U$600–1000	Human
Asia	Epidiolex	CBD	Epilepsy (restrict usage)	U$1000	Human
Asia (India)	Clasepi	CBD (synthetic)	Severe epilepsy (Dravet syndrome, Lennox–Gastaut syndrome, and tuberous sclerosis complex)	U$110–120	Human
Asia (India)	Cannepsy	CBD (synthetic)	Severe epilepsy (Dravet syndrome, Lennox–Gastaut syndrome, and tuberous sclerosis complex)	U$110–120	Human
Asia (India)	MSN CBD	CBD (synthetic)	Severe epilepsy (Dravet syndrome, Lennox–Gastaut syndrome, and tuberous sclerosis complex)	U$105	Human
Asia (India)	Cannazo Pet Oil	Full spectrum (Ayurvedic)	Anxiety, dermatological conditions, inflammation, and pain in animals	U$24	Veterinary
Asia (India)	Caneura Oral Solution Delicious Strawberry	CBD (Synthetic)	Epilepsy (Dravet syndrome and Lennox–Gastaut syndrome)—patients aged ≥ 2 years	U$90–113	Human
Asia (India)	Hempstrol Pet CBD Oil	Full spectrum	Anxiety, pain, seizures, and general well-being in animals	U$60–180	Veterinary
Asia (India)	Hempstrol Full Spectrum CBD Soft Gels	Full spectrum	Chronic pain and inflammation	U$20–115	Experimental veterinary usage (off-label)
Asia (India)	Cannazo CBD Oil	CBD (Ayurvedic)	Anxiety, pain, and insomnia	U$30–100	Experimental veterinary usage
Asia (India)	Boheco CBD Oil	CBD or full Spectrum	Anxiety and mild epilepsy	U$50–150	Experimental veterinary usage
Asia (India)	Cannarma	CBD	Stress, pain, and sleep disorders	U$40–100	Human
Asia (India)	Canna Balance	CBD	Chronic pain, anxiety, stress, insomnia, inflammation, and metabolic regulation	U$54	Human
Asia (India)	Cannavedic	THC + CBD (Ayurvedic)	Chronic pain, anxiety, inflammation, insomnia, and loss of appetite	U$100–180	Experimental veterinary usage
Asia (India)	Cannavedic	THC + CBD (Ayurvedic)	Severe pain, muscle spasms, insomnia, and palliative care	U$80–200	Experimental veterinary usage
Asia (India)	Cannazo	THC + CBD (Ayurvedic)	Severe chronic pain, edema, inflammation, and palliative care	U$80–200	Experimental veterinary usage
South America (Brazil)	Cannabidiol	CBD (Isolated)	Epilepsy, anxiety, chronic pain	U$15–350	Human
South America (Brazil)	Cannabidiol	CBD (Isolated)	Epilepsy, anxiety	U$100–250	Human
South America (Brazil)	Cannabidiol	CBD (Isolated)	Refractory epilepsy	U$150–300	Human
South America (Brazil)	Cannabidiol	CBD (Isolated)	Anxiety, pain, epilepsy	U$120–280	Human
South America (Brazil)	Cannabidiol	CBD (Isolated)	Epilepsy	U$150–300	Human
South America (Brazil)	Cannabidiol	CBD (Isolated)	Anxiety, pain	U$100–250	Human
South America (Brazil)	Cannabidiol Greencare	CBD (Isolated)	Anxiety, pain	U$100–250	Human
South America (Brazil)	Cannabidiol	CBD (Isolated)	Mild anxiety, pain	U$80–200	Human
South America (Brazil)	Cannabidiol)	CBD (Isolated)	Epilepsy, pain	U$120–300	Human
South America (Brazil)	Cannabidiol	CBD (Isolated)	Epilepsy	U$150–300	Human
South America (Brazil)	Cannabidiol	CBD (Isolated)	Mild anxiety	U$80–180	Human
South America (Brazil)	Cannabis Extract	CBD + THC	Chronic pain, palliative care	U$150–400	Human
South America (Brazil)	Cannabis Extract	CBD + THC	Pain, spasticity	U$150–400	Human
South America (Brazil)	Cannabis Extract	CBD + THC	Chronic pain	U$150–400	Human
South America (Brazil)	Cannabis Extract	CBD + THC	Pain, epilepsy	U$120–350	Human
South America (Brazil)	Cannabis Extract	CBD + THC	Pain, inflammation	U$120–300	Human
South America (Brazil)	Cannabis Extract	CBD + THC	Severe pain, palliative care	U$150–400	Human
South America (Brazil)	Cannabis Extract	CBD + THC	Mild anxiety	U$80–200	Human

## Data Availability

No new data were created or analyzed in this study.

## Abbreviations

The following abbreviations are used in this manuscript:

2-AG	2-arachidonoylglycerol
AI	Artificial Intelligence
AYUSH	Ayurveda, Yoga and Naturopathy, Unani, Siddha and Homoeopathy
CBD	Cannabidiol
CBDA	Cannabidiolic acid
CBG	Cannabigerol
CBN	Cannabinol
CBPI	Canine Brief Pain Inventory
CB1	Cannabinoid receptor type 1
CB2	Cannabinoid receptor type 2
CNS	Central nervous system
ECS	Endocannabinoid system
EE	Encapsulation efficiency
FAAH	Fatty acid amide hydrolase
FDA	Food and Drug Administration
HIV	Human immunodeficiency virus
IASP	International Association for the Study of Pain
LAs	Local anesthetics
LUV	Large unilamellar vesicles
MAGL	Monoacylglycerol lipase
NDPS	Narcotic Drugs and Psychotropic Substances
NE	Nanoemulsion
NLC	Nanostructured lipid carrier
NSAIDs	Nonsteroidal anti-inflammatory drugs
PDI	Polydispersity index
PIS	Pain interference score
PNS	Peripheral nervous system
PSS	Pain severity score
QoLI	Quality of life index
RDC	Collegiate Board Resolution
THC	Δ9-tetrahydrocannabinol
THCA	Tetrahydrocannabinolic acid
TSC	Tuberous sclerosis complex
USD	United States dolar
ZP	Zeta potential
